# The Efficacy of Continuous Serratus Anterior and Erector Spinae Plane Blocks vs Intercostal Nerve Block in Uniportal-Vats Surgery: A Propensity-Matched Prospective Trial

**DOI:** 10.3390/jcm13020606

**Published:** 2024-01-21

**Authors:** Dania Nachira, Giovanni Punzo, Giuseppe Calabrese, Flaminio Sessa, Maria Teresa Congedo, Giovanna Beccia, Paola Aceto, Khrystyna Kuzmych, Chiara Cambise, Carolina Sassorossi, Adriana Nocera, Alessia Senatore, Maria Letizia Vita, Elisa Meacci, Liliana Sollazzi, Stefano Margaritora

**Affiliations:** 1 Department of General Thoracic Surgery, Fondazione Policlinico Universitario A. Gemelli-IRCCS, Università Cattolica del Sacro Cuore, Largo A. Gemelli, 8, 00168 Rome, Italymariateresa.congedo@policlinicogemelli.it (M.T.C.); khrystyna.kuzmych01@icatt.it (K.K.); carolina.sassorossi01@icatt.it (C.S.); adriana.nocera01@icatt.it (A.N.); stefano.margaritora@policlinicogemelli.it (S.M.); 2 Department of Anesthesiology and Intensive Care Medicine, Fondazione Policlinico Universitario A. Gemelli-IRCCS, Università Cattolica del Sacro Cuore, 00168 Rome, Italy; giovanni.punzo@policlinicogemelli.it (G.P.); giovanna.beccia@policlinicogemelli.it (G.B.); paola.aceto@policlinicogemelli.it (P.A.); chiara.cambise@policlinicogemelli.it (C.C.); liliana.sollazzi@policlinicogemelli.it (L.S.)

**Keywords:** VATS surgery, postoperative analgesia, fascial plane blocks, intercostal nerve blocks, erector spinae plane block

## Abstract

Background: To evaluate the analgesic efficacy of continuous erector spinae plane block(c-ESPB) and serratus anterior plane block(c-SAPB) versus the intercostal nerve block (ICNB) in Uniportal-VATS in terms of pain control, drug consumption, and complications. Methods: Ninety-three consecutive patients, undergone one of the three peripheral nerve blocks after Uniportal-VATS, were prospectively enrolled. A 1:1 propensity score matching was used to minimize bias. Results: C-ESPB and c-SAPB groups had no difference in morphine request upon awakening compared to ICNB. A higher VAS-score was recorded in c-ESPB compared to ICNB in the first 12 h after surgery. A significantly lower consumption of paracetamol in II postoperative day (p.o.d.) and tramadol in I and II p.o.d. was recorded in the c-ESPB group compared to the ICNB group. A higher dynamic VAS score was recorded at 24 h and 48 h in the ICNB group compared to the c-SAPB. No difference was found in safety, VAS-score and drug consumption between c-ESPB and c-SAPB at any given time, except for a higher tramadol request in c-SAPB in II p.o.d. Conclusions: C-ESPB and c-SAPB appear to have the same safety and analgesic efficacy when compared between them and to ICNB in Uniportal-VATS approach. C-ESPB showed a delayed onset of analgesic effect and a lower postoperative drug consumption compared to ICNB.

## 1. Introduction

Postoperative pain management has major significance in thoracic surgery, impacting patient recovery and reducing the incidence of cardio-pulmonary complications. Uniportal video-assisted thoracic surgery (Uniportal-VATS) is reported to be the least painful and minimally invasive thoracic approach owing to its involvement of only one intercostal space and the relatively anterior location of the incision (where the intercostal spaces are wider, thereby minimizing the risk of intercostal nerve injury) [1]. However, postoperative pain in patients undergoing Uniportal-VATS can, in certain cases, be intense, with the chest tube itself being a potential source of postoperative pain and discomfort [2,3].

Thoracic epidural analgesia (TEA) and thoracic paravertebral block (TPVB) have been traditionally considered the gold standard for postoperative pain control in thoracic surgery [4]. However, both TEA and TPVB carry the risk of serious side effects and complications, requiring specific expertise for their administration and an appropriate discontinuation of anticoagulants [4].

Intercostal nerve block (ICNB) has emerged as one of the most widely used alternatives to TEA and TPVB, often employed for postoperative pain management after Uniportal-VATS across various centers [5,6]. Despite its frequent use, ICNB has limitations in terms of its duration, requiring multiple injections in the thoracic wall, potentially increasing the risk of complications, and may fail to achieve optimal postoperative analgesia. Consequently, clinicians have explored possible alternative options for the ICNB, such as the Serratus Anterior Plane Block (SAPB) and the Erectus Spinae Plane Block (ESPB).

The SAPB was first described by Blanco in 2013 [7]. It provides anterolateral analgesia of the chest wall by blocking the lateral cutaneous branches of T2-T9, the long thoracic nerve (LTN), thoracodorsal nerve, and the intercostobrachial nerve.

Introduced more recently by Forero in 2016 [8], the ESPB is a posterior fascial block that provides somatic analgesia to both cutaneous and deeper musculoskeletal tissues by blocking the dorsal and ventral branches of thoracic spinal nerves (T2-T10) [8,9]. Moreover, this block is supposed to induce visceral anesthesia and modulate sympathetic activity [10,11]. 

Single-shot ESPB and SAPB were recently described in thoracic surgery, including Uniportal-VATS [12,13,14,15], but their efficacy remains still unclear. Furthermore, the American Pain Society Guidelines [16] strongly recommend the use of continuous peripheral regional analgesia techniques over single injections for postoperative pain management. 

Hence, the main aim of this study was to compare the continuous ESPB (c-ESPB, a posterior fascial block) and the continuous SAPB (c-SAPB, a lateral fascial block) against the well-established ICNB in patients undergoing Uniportal-VATS, in order to evaluate differences in postoperative pain control, administration of analgesic drugs, and occurrence of complications. 

## 2. Materials and Methods

### 2.1. Ethical Statement

This study was approved by the Ethical Committee (Università Cattolica del Sacro Cuore) in March 2021 (Prot.ID 3921), registered on Clincaltrial.gov (identifier: NCT04892901) and therefore conducted in accordance with the ethical standards of the Declaration of Helsinki and its later amendments. All patients provided informed consent to participate in the study, ensuring their clinical data were treated anonymously. 

### 2.2. Study Design

The study was a prospective, single-center, nonrandomized trial. From January 2022 to June 2023, the clinical data of 93 consecutive patients who received a peripheral nerve block after lung surgery using the Uniportal-VATS approach was collected prospectively. In this study, data and results were reported using the Strengthening the Reporting of Observational Studies (STROBE) checklist.

The inclusion criteria of the study were: lung resections in Uniportal-VATS, adult patients (age ≥ 18 years), body-mass index (BMI) < 30 kg/m^2^, and signed informed consent. Exclusion criteria involved associated pleural, diaphragm, or chest wall resections, active anticoagulant therapy, severe scoliosis, previous thoracic surgery, any painkiller allergy, chronic opioid usage, single-kidney patients, patients with liver disease, or patients suffering from psychiatric or neurodegenerative diseases.

Surgery was conducted under general anesthesia, employing single-lung ventilation and positioning the patients in lateral decubitus. In all cases, the average surgical incision was 4–5 cm in length, completely muscle-sparing, and located along the IV-V intercostal space on the middle axillary line. To prevent contamination from tumors or infections, only a wound protector was used during surgery, without trocars or rib retractors. At the end of surgery, a 28-Fr chest tube was placed through the same incision and secured on the posterior side [2]. 

### 2.3. Loco-Regional Blocks

To prevent any timing-related bias associated with block execution, all blocks were administered immediately after surgery, while patients were under general anesthesia.

The ICNB was carried out by surgeons injecting 4 mL of 0.5% ropivacaine into the intercostal spaces (III–VII) under thoracoscopic guidance before closing the surgical incision. This was supplemented with postoperative intravenous tramadol (400 mg/48 h) administration via an elastomeric pump to extend postoperative analgesia, since the ICNB cannot be performed continuously. 

The continuous-SAPB (c-SAPB) was performed at the end of surgery (by 3 skilled surgeons) before closing muscle layers and skin via surgical catheterization of the superficial serratus fascial plane (if it remained intact during surgical incision). Under direct visualization, a 18G Tuohy needle was inserted by the surgeon into the posterior side of the chest wall, on the same intercostal space as the surgical incision but 8–10 cm away from it. The tip was directed to reach the superficial fascia of the serratus anterior muscle. Then, 3–4 mL of saline solution was injected to open the virtual fascial plane between the serratus anterior and the latissimus dorsi. The correct positioning of the needle tip was confirmed by the surgeon observing a slight swelling of the serratus anterior superficial fascia between the muscle layers through the Uniportal-VATS incision. Then, an epidural catheter was inserted approximately 8 cm through the needle into the space created by the saline solution under the fascia. Throughout the procedure, the surgeon had continuous visual access through the Uniportal-VATS incision and checked the tip of the epidural catheter, which was clearly visible in transparency through the superficial fascia of the anterior serratus.

The continuous ESPB (c-ESPB) was administered by two anesthesiologists with extensive experience in ultrasound-guided fascial blocks. The erector spinae muscles were identified at the T5 level, approximately 2–3 cm away from the spinal processes. Subsequently, an 18G Tuohy needle was inserted in the cranial-caudal direction using an in-plane technique until bony contact with the T5 transverse process was obtained. Then, 3–4 mL of saline was injected to dissect the erector spinae from the transverse processes, followed by the threading of an epidural catheter through the needle. 

In both c-SAPB and in c-ESPB procedures, 20 mL of 0.5% ropivacaine were injected into the corresponding fascial plane. Additionally, a 5 mL/h elastomeric pump filled with 240 mL of 0.2% ropivacaine was connected to the epidural catheter to ensure a continuous 48-h infusion of the local anesthetic.

The type of block used was determined on a case-by-case basis by the surgical and anesthesiology teams. This decision was guided by the experience and expertise in peripheral blocks of the attending operators (surgeons/anesthesiologists), along with other surgical factors (such as disruption or not of the superficial fascial plane of serratus muscle/intercostal fascial planes during surgery), or logistic variables (availability of materials and ultrasound equipment).

Consequently, based on the type of block performed, patients were prospectively enrolled in the following 3 groups: c-SAPB group (study group 1), c-ESPB group (study group 2), and the ICNB group (ICNB+ postoperative + IV tramadol, control group).

Immediately after extubation, patients were transferred to the postanesthetic recovery room (PAR) and monitored for approximately 2–4 h. The main parameters for discharging patients from PAR and transferring them to the surgical ward included well-controlled postoperative pain (VAS scores <3), no sign of bleeding or severe air leakage from the chest tube, an Aldrete score = 10, and no radiological complications at the postoperative chest X-ray. 

An array of clinical and surgical variables were prospectively recorded per patient across the groups. These variables encompassed gender, age, smoking habits, BMI, chronic obstructive pulmonary disease (COPD), any cardiovascular disease, the American Society of Anaesthesiologists (ASA) score, side of surgery, type of surgery and surgical time, and lung functionality (paO_2_, PaCO_2_, FEV1%, and FVC%). Additionally, other recorded variables involved time of block administration, postoperative pain (measured by the visual analogue scale (VAS) score) at 0, 2, 8, 12, 24, and 48 h at rest (static VAS score), and while coughing (dynamic VAS score). Furthermore, VAS evaluations during chest tube removal, at 2 h postremoval, and after 12 weeks postsurgery were included to assess the potential onset of chronic postsurgical pain (CPSP). Details regarding analgesic drug usage and total morphine consumption within the initial 48 h after surgery were also documented. Finally, any type of complications (block-related: chest wall hematoma, catheter dislodgement, catheter discomfort, nausea, paraesthesia, or not block-related: atrial fibrillation, lung atelectasis) were reported and analysed.

### 2.4. Pain Management

A standardized postoperative pain control protocol was administered to all patients. Twenty minutes before extubation, all patients were administered intravenous 1000 mg paracetamol as the loading dose for analgesia. Subsequently, for the first 48 h postsurgery, all patients received intravenous 1000 mg paracetamol three times per day (with a maximum 3000 mg/24 h) unless they reported no pain (VAS scores = 0). In cases of static or dynamic VAS scores 2–4, ketorolac 30 mg was administered as rescue therapy (repeatable every 8 h, up to a maximum of 90 mg/24 h). VAS scores higher than 4 were managed with a 2 mg morphine bolus (repeatable every 30 min up to a maximum of 10 mg) while in PAR or with tramadol 50 mg (repeatable every 8 h up to a maximum of 150 mg) if the patient had already been transferred to the surgical ward.

### 2.5. Primary and Secondary Outcomes 

The primary outcome was defined as the level of pain experienced in the first 48 h after surgery, evaluated by the static and dynamic VAS scores, as well as the total daily drug usage as rescue therapy. Secondary outcomes included the block execution time, any complications block-related or otherwise, pain during chest tube removal, and the onset of CPSP.

### 2.6. Sample Size

The trial was designed to test the hypothesis that the levels of postoperative pain in the study groups were not superior when compared to the control group. An “a priori” analysis was performed given an effect size of 0.5, a power of 90%, and a type I error of 5% (α). A 10% dispersion of patients at follow-up or due to catheter dislodgment was also considered. Consequently, 25 patients per group were estimated. G*Power (ManchesterMetropolitan University, Manchester, UK) was used for sample size calculation [17].

### 2.7. Statistical Analysis

Categorical variables were expressed as numbers (%), while continuous variables were expressed as mean ± standard deviation, or medians for non-normally distributed data. Categorical variables were compared by the Chi-square test; continuous variables by the independent-sample Student’s *t*-test or the Mann–Whitney U-test, if normally or non-normally distributed. The normality of the data distribution was assessed with the Shapiro–Wilk test. One-way analysis of variance (ANOVA) for repeated measures was used to determine the differences in VAS score between groups at different times. Initially, the ICNB group was compared with the c-SAPB group, followed by comparison with the c-ESPB group. Moreover, the c-SAPB and the c-ESPB were also compared with one another. To overcome the biases stemming from nonrandomized enrolment, a 1:1 propensity score was generated using the nearest neighbour matching method. This approach was aimed at balancing the baseline characteristics of the two compared groups in each round of comparison. The variables included in the propensity score model were age, gender, smoking habits, COPD, cardiovascular diseases, diabetes mellitus, ASA score, type of lung resection, side, surgical time, and lung functionality. A *p* < 0.05 was considered statistically significant. Statistical analysis was performed using IBM SPSS Statistics for Macintosh, Version 25.00 (Armonk, NY, USA).

## 3. Results

Out of the 93 patients enrolled in the study, 40 patients were in the ICNB group, 27 were in the c-SAPB group, and 26 were in the c-ESPB group. 

To reduce any recruitment bias, a 1:1 propensity score-matched analysis was conducted, resulting in 20 eligible patients from the two compared groups in every round of comparison.

The main clinical and surgical characteristics of the patients within in the respective matched groups are reported in Table 1A,B.

**Figure 1:**
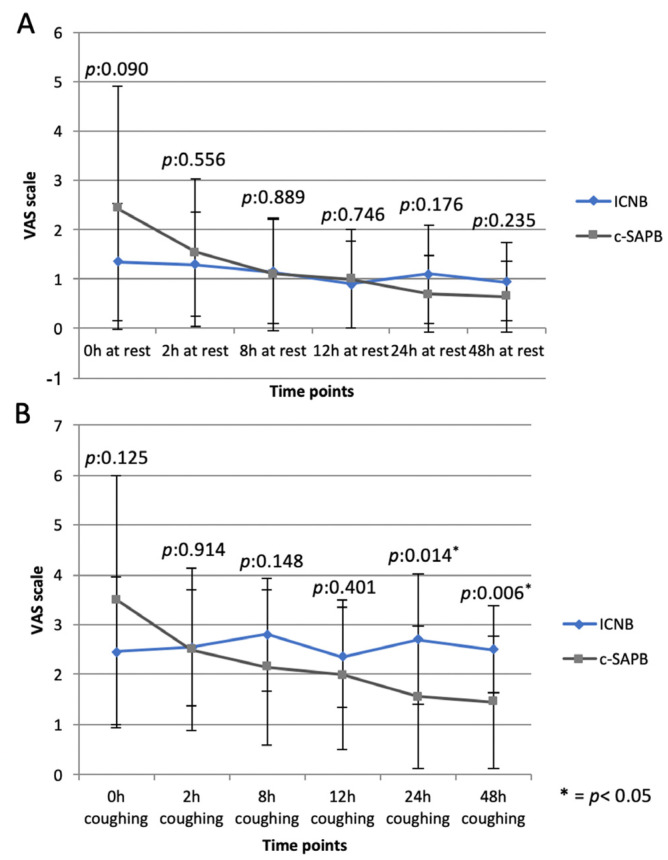
Static (**A**) and dynamic (**B**) VAS scores in the c-SAPB and ICNB groups at different time points. * = *p* < 0.05.

When comparing the c-SAPB group to the ICNB (control group), no difference was recorded in terms of block execution time (2.53 ± 0.88 vs. 3.85 ± 2.52 min, p:0.09) and morphine request in PAR for VAS score > 4 (p:0.212). However, a higher dynamic VAS score was recorded at 24 h (p:0.014) and 48 h (p:0.006), as well as during chest tube removal (p:0.022) in the ICNB group compared to the c-SAPB (Figure 1, Table 2). 

Additionally, no difference was observed in terms of total amount and type of painkillers taken in I and II postoperative days, except for higher paracetamol consumption in c-SAPB in I p.o.d. (p:0.028), as outlined in Table 2. No complications related to the execution of the block were observed in any group. There was only one case (5%) of lung atelectasis in the ICNB group. 

When comparing the c-ESPB group with the ICNB one, no differences were recorded in block execution time (2.42 ± 0.83 vs. 3.75 ± 1.23 min, p:0.130), morphine request upon awakening in PAR for VAS > 4 (p:0.429), or total morphine amount (0.113), Table 3. 

**Figure 2:**
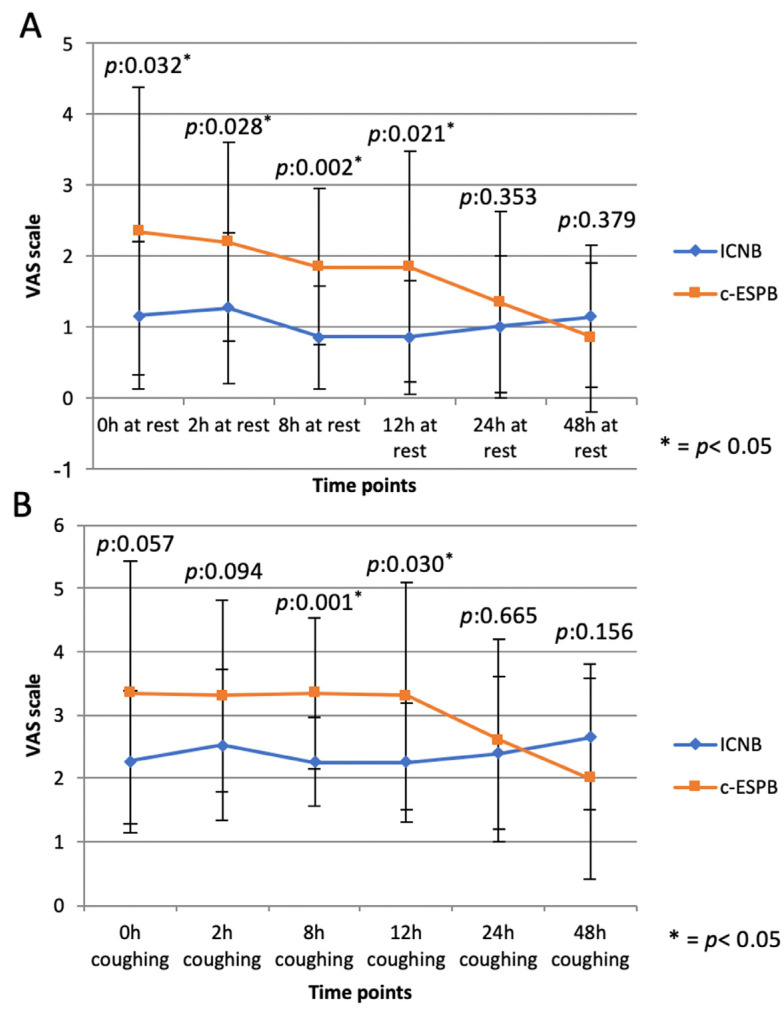
Static (**A**) and dynamic (**B**) VAS scores in the c-ESPB and ICNB groups at different time points. * = *p* < 0.05.

A higher VAS score was recorded in the c-ESPB compared to the ICNB at 0 h (static: p:0.032), 2 h (dynamic: p:0.028), 8 h (static: p:0.002; dynamic: p:0.001), and 12 h (static:p:0.021; dynamic:p:0.030); no difference was found at 24 h and 48 h (Figure 2).

There was only a trend toward significance for a higher level of pain during chest tube removal in the ICNB (3.06 ± 1.21 vs. 2.35 ± 1.34, p:0.072), Table 3.

A significantly higher request for paracetamol on the II postoperative day (p:0.005) and on-demand tramadol consumption on I (p:0.05) and II p.o.d. (p:0.012) were recorded in the ICNB compared to the c-ESPB, Table 3.

No differences were noted between the two groups in terms of CPSP, neuralgia, and complications related to or independent of the blocks, which were null. 

**Figure 3:**
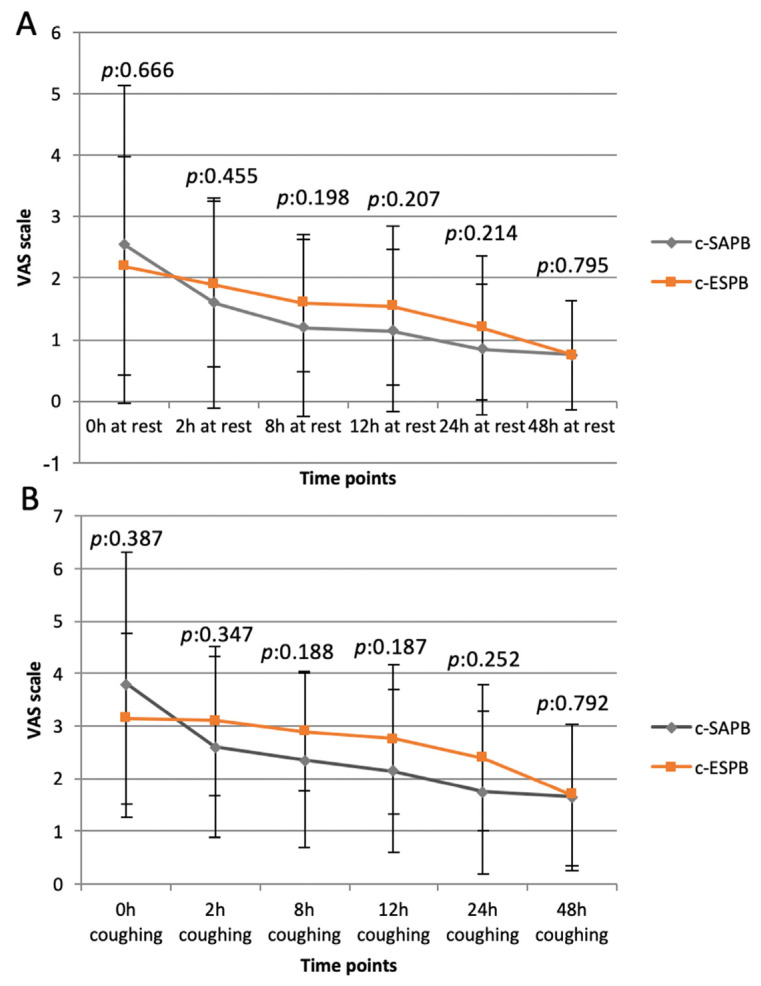
Static (**A**) and dynamic (**B**) VAS scores in c-SAPB and c-ESPB groups at different time points.

Interestingly, when comparing the c-ESPB and c-SAPB groups, no significant differences were found in VAS scores (Figure 3) or drug requirement at any given time, except for a higher on-demand tramadol consumption in the c-SAPB group on II p.o.d. (p:0.012). 

No differences were recorded in the level of pain during chest tube removal (p:0.748) or in the incidence of CPSP, which were null. Moreover, no complications, related to the blocks or otherwise, were recorded in any group (Table 4).

## 4. Discussion

The efficacy and role of peripheral fascial blocks in managing postoperative pain in patients undergoing Uniportal-VATS remain highly debated topics [18,19]. Both SAPB and ESPB, when performed via the “single-shot” technique, provide pain relief lasting for approximately 12–24 h postsurgery, but the duration of postoperative pain in patients undergoing Uniportal-VATS can last longer, up to 72 h. Consequently, longer-lasting blocks, such as c-SAPB and c-ESPB, may be recommended for these patients.

To our knowledge, only two studies evaluated the effects of c-SAPB in patients undergoing Uniportal-VATS [20,21]. In the first of these, Allain et al. compared the analgesic efficacy of systemic analgesia alone or in combination with c-TPVB, single-shot SAPB, or c-SAPB [20]. The authors concluded that combining systemic analgesia and c-SAPB might present some advantages, such as lower consumption of opioid drugs compared to the other analgesic techniques studied [20]. However, limitations such as small sample size and the absence of randomization or propensity score matching in Allain’s study weaken their results and suggest a need for further verification. In the second study, c-SAPB was used in Uniportal-VATS with the main aim of verifying whether the addition of dexamethasone to the anesthetic mixture administered continuously into the interfascial plane underneath the serratus anterior muscle increases the duration of the block or not [21]. Therefore, in that study [21], c-SAPB was not compared to other nerve block or analgesic techniques, and no evidence was provided to establish whether c-SAPB is more or less effective than other regional anesthesia techniques in patients undergoing Uniportal-VATS.

In the present study, the c-SAPB was compared with a routine analgesic protocol used at our center in patients undergoing Uniportal-VATS and of proven efficacy in the first 48 h after surgery: single-shot ICNB and continuous intravenous infusion of tramadol for 48 h via elastomeric pump. According to our results, c-SAPB appears to be an effective block in Uniportal-VATS, realizing mean pain scores below 4 during the entire study period (Figure 1). Static and dynamic VAS scores were similar to those recorded at the same time points in the ICNB group, except for lower dynamic VAS scores at 24 h (p:0.014) and 48 h (p:0.006) postsurgery (Figure 1). This would suggest that c-SAP has greater effectiveness against movement-related pain, likely due to the block of the LTN. Indeed, the LTN lies on the anterior surface of the serratus anterior muscle and is completely or partially blocked when SAPB is performed by injecting local anesthetic into the potential space between the latissimus dorsi and the serratus anterior muscle [22], as in our series. The LTN innervates the serratus anterior muscle, whose contractions after VATS may irritate the injured intercostal muscles and increase their tension, thereby aggravating pain associated with movement [22].

Regarding ESPB, the few reports currently available on the use of this block in Uniportal-VATS refer exclusively to the single-shot technique, with no evaluation of the continuous approach in this kind of surgery so far. In 2020, for the first time, Liu et al. [12] showed that ultrasound-guided single-shot ESPB might reduce perioperative opioid consumption and plasma cytokine levels in Uniportal-VATS. Recently, the ESPB used as a “rescue block” for uncontrolled pain upon awakening from Uniportal-VATS [23] was also described by our group. However, Sertcakacilar et al. [13] reported lower analgesic efficacy and a higher morphine request in patients who underwent ultrasound-guided one-shot ESPB in Uniportal-VATS, when compared with TVPB. 

In 2021, Lonqvuist et al. [24] questioned the analgesic effect of ESPB between the mid-axillary and the parasternal lines due to the lack of proven mechanisms of action that could explain how this posterior nerve block influences the anterior part of the intercostal nerves. According to the authors’ opinion [24], there is insufficient evidence demonstrating that the analgesic effects of the ESPB may be partly linked to the plasma local anesthetic levels usually associated with fascial blocks. This uncertainty arises because the ESPB has often been compared to either a placebo or standard systemic analgesic regimens, which typically shows low or negligible efficacy. As a result, Lonqvuist [24] strongly advocated for further high-quality studies in which single-shot and/or c-ESPB are compared to other well-established analgesic techniques. 

Therefore, in the present study, the analgesic efficacy of c-ESPB in the first 48 h after surgery was compared with that of ICNB, considered one of the most effective nerve blocks in thoracoscopic surgery. Both c-ESPB and ICNB were performed with the same timing (immediately after surgery) and by using the same initial bolus of local anesthetic (20 mL of 0.5% ropivacaine). Furthermore, since ICNB cannot be administered continuously, this block was associated with a continuous infusion of tramadol. In our experience, this approach effectively extends postoperative analgesia in patients who undergo single-shot ICNB, providing pain relief for up to 48–72 h following surgery. 

According to our results, static and dynamic VAS scores in the c-ESPB group appeared to be higher in the early postoperative period (0–12 h) compared to ICNB, although comparable results were observed during coughing at 0 and 2 h and under all conditions at 24 h and 48 h (Figure 2). These results seem to be in contrast with those reported in a recent RCT by Chaudhary et al. [25] involving patients undergoing triportal or robotic surgery. These authors recorded a significant improvement in static VAS scores in the first 24 h after surgery and a reduction in PAR length of stay in the single-shot ESPB group when compared to ICNB performed by surgeons at the end of surgery [25]. However, the static VAS scores reported in the Chaudhary’s study on I p.o.d. were comparable to those recorded in the present study. Moreover, the ESPB in the Chaudhary’s study was performed in two posterior approaches (and not in Uniportal-VATS, which is an antero-lateral approach) and preoperatively, deemed to be the most effective way of providing postoperative analgesia with this block [26]. Finally, a higher ropivacaine volume was used in the Chaudary’s study for the initial bolus (40 mL [25] vs. 20 mL in our series), as in several series that demonstrated a high ESPB efficacy in the early postoperative period [12,27,28,29,30].

In our study, when the c-SAPB and the c-ESPB were compared, no differences in mean static and dynamic VAS scores were found between the two study groups. These results differed from those of Zhang’s et al. [14], who reported a superior analgesic effect of the ESPB when compared to SAPB during the first 48 h after surgery in patients undergoing Uniportal-VATS.

It is interesting to note that patients who received fascial blocks seemed to have better pain control during chest tube removal compared to those receiving ICNB (VAS score: 1.95 ± 1.27 in the c-SAPB group vs. 3.00 ± 1.15 in the ICNB group, p:0.022; 2.35 ± 1.34 in the c-ESPB group vs. 3.06 ± 1.21 in the ICNB group, p:0.072), which occurs 3 days after surgery on average (mean: 3.42 ± 2.33 days), 24 h after the removal of the fascial catheter. These findings might suggest long-lasting pain modulation and analgesia in continuous fascial block groups. This effect could potentially contribute to the reduction in the incidence of CPSP, which was null in our series.

With regard to the type and overall quantity of painkillers taken in the first 48 h after surgery, our results suggest a comparability between the c-SAPB and the ICNB groups, except for a higher paracetamol consumption in the c-SAPB group on I p.o.d. (p:0.028, Table 2). Conversely, in the c-ESPB group, the consumption of painkillers during the first 48 h appears to be significantly reduced when compared to the ICNB group (Table 3). This occurred mainly on the second postoperative day, during which, in the c-ESPB group, both paracetamol (p:0.005, Table 3) and on-demand tramadol consumption (p:0.012, Table 3) were significantly lower than in the ICNB group. Moreover, on-demand tramadol consumption was lower in the c-ESPB than in the ICNB group even on the first p.o.d. (p:0.005, Table 3). The advantages in terms of lower consumption of opioid drugs in the c-SAPB and especially in the c-ESPB group compared to the control group (ICNB + continuous IV infusion of tramadol) would become even more evident (and statistically significant both on I and II p.o.d. for both fascial blocks) if, aside from considering the consumption of opioids on request, the dose of tramadol (200 mg/die) necessary to guarantee patients undergoing ICNB an adequate duration of analgesic coverage was also considered. However, the main aim of the study was to evaluate the effectiveness of the two fascial blocks in terms of total daily drug usage as rescue therapy, compared to the standard established practice of ICNB+ continuous IV infusion of 200 mg of tramadol per day.

In general, our results seem to confirm those of a recent pilot randomized controlled trial on patients undergoing minimally invasive thoracoscopic surgery (not Uniportal-VATS), wherein c-ESPB appeared to reduce overall opioid consumption within the first 48 h after surgery in comparison to single-shot ICNB [31].

Both fascial blocks and the ICNB showed no difference in morphine request in PAR for VAS score >4 and in total morphine amounts when compared among themselves in our study. All three types of blocks appeared easy and safe to be performed in Uniportal-VATS, requiring comparable execution times (range: 1.58–6.02 min) and the absence of related complications, such as hematomas, catheter dislodgment or discomfort, nausea, or paraesthesia. Although a sympathetic modulation was described in ESPB [8,9] our study failed to point out any postoperative outcome related to sympathetic effects, such as atrial fibrillation or lung atelectasis. This is probably due to the relatively small sample size.

Overall, the differences that emerged between the ICNB and the two fascial blocks evaluated, although worthy of further investigation, are few and probably hold little clinical relevance. Even considering the total dosage of opioids administered in the ICNB group (also accounting for a part of the total of 200 mg per day infused IV to maintain postoperative analgesia), the daily consumption of tramadol in this group was well below the upper limit for this drug (400 mg/day), and no opioid-related adverse effects were recorded in our series. Therefore, in our opinion, ICNB could still remain the block of choice for patients undergoing Uniportal-VATS, while reserving c-SAPB and c-ESPB for patients undergoing more painful surgical approaches or as a rescue measure for Uniportal-VATS patients experiencing breakthrough pain despite ICNB, requiring large amounts of opioids.

### Limitations and Points of Strength

This study has several limitations, including its monocentric and nonrandomized nature. In addition, a quite subjective index (VAS score) was used to evaluate the analgesic effect; however, the evaluation was always performed by the same two thoracic surgeons to avoid potential variability. Additionally, no evaluation of the dermatomal level for analgesia was undertaken, such as by a pinprick test, as all procedures were performed under general anesthesia. Lastly, the “on-demand” postoperative drugs administered to patients may represent a confounding factor when comparing groups among them.

However, this study also has some points of strength. First, it was a prospective trial, with a sample size evaluated based on the primary outcome. Second, a propensity-match analysis was conducted to compensate for possible selection biases. Third, this is the first study that assesses the feasibility and effectiveness of c-ESPB and c-SAPB in patients undergoing an anterolateral surgical approach, such as Uniportal-VATS. Fourth, both c-SAPB and c-ESPB were compared to ICNB, a block previously proven effective in Uniportal-VATS surgery. Fifth, all nerve blocks were performed postoperatively to reduce any bias related to different execution times, and each was performed by experienced operators. Lastly, all patients were assessed for the onset of CPSP not only within the first 48 h after surgery but also at the time of chest tube removal and 12 weeks after the procedure.

## 5. Conclusions

In conclusion, both c-SAPB and c-ESPB seem feasible, effective, and easy to perform in patients undergoing Uniportal-VATS, representing valid options for postoperative pain management in these patients. Specifically, c-SAPB exhibits comparable safety and analgesic efficacy to ICNB in Uniportal-VATS. Meanwhile, c-ESPB appears to be less effective than ICNB in reducing early postoperative pain (0–12 h after surgery) but shows potential for reducing analgesic drug consumption in the postoperative period when compared to ICNB. Both continuous fascial blocks appear to have a long-lasting modulating action on postoperative pain, persisting even after the conclusion of the continuous local anesthetic infusion.

However, further studies involving larger cohorts are necessary to confirm our results, evaluate the analgesic effects of continuous fascial blocks in Uniportal-VATS, and explore the potential effects of the c-ESPB in terms of visceral analgesia and sympathetic modulation.

## Figures and Tables

**Table 1:** Preoperative characteristics after 1:1 propensity score matching groups ICNB and c-SAPB (**A**) and groups ICNB and c-ESPB (**B**).

(A)			
	ICNB (#20)	C-SAPB (#20)	*p*
Gender (male)	8 (40.0%)	8 (40.0%)	1.00
Age (years)	64.45 ± 12.82	64.70 ± 12.36	0.950
Smoker	4 (20.0%)	4 (20.0%)	1.00
BMI	25.93 ± 3.97	26.03 ± 4.12	0.940
COPD	6 (30.0%)	4 (20.0%)	0.465
Diabetes mellitus	3 (15.0%)	2 (10.0%)	0.633
Cardiovascular diseases	9 (45.0%)	9 (45.0%)	1.00
ASA SCORE	2.40 ± 0.50	2.25 ± 0.44	0.324
Side (right)	9 (45.0%)	10 (50.0%)	0.752
Surgical time (min)	99.50 ± 45.63	96.70 ± 36.96	0.832
Wedge/lobectomy	15 (75.0%)/3 (15.0%)	15(75.0%)/4 (20.0%)	0.788
PaO_2_	82.93 ± 4.71	86.65 ± 8.84	0.514
PaCO_2_	39.05 ± 1.69	40.95 ± 0.71	0.210
FEV1%	117.00 ± 2.83	86.50 ± 34.65	0.340
FVC%	97.00 ± 19.79	95.00 ± 28.28	0.942
**(B)**			
	**ICNB (#20)**	**C-ESPB (#20)**	***p***
Gender (male)	7 (35.0%)	7 (35.0%)	1.00
Age (years)	64.20 ± 13.07	64.20 ± 11.67	1.00
Smoker	0	1 (5.0%)	0.311
BMI	25.83 ± 3.96	26.16 ± 2.04	0.838
COPD	5 (25.0%)	3 (15.0%)	0.429
Diabetes mellitus	2 (10.0%)	2 (10.0%)	1.00
Cardiovascular diseases	7 (35.0%)	7 (35.0%)	1.00
ASA SCORE	2.25 ± 0.72	2.25 ± 0.44	1.00
Side (Right)	11 (55.0%)	12 (60.0%)	0.749
Surgical time (min)	94.70 ± 51.61	102.42 ± 46.01	0.625
Wedge/lobectomy	6(60.0%)/13 (65.0%)	7(35.0%)/13 (65.0%)	0.584
PaO_2_	87.43 ± 6.29	61.75 ± 30.20	0.147
PaCO_2_	37.90 ± 2.03	58.00 ± 29.43	0.222
FEV1%	113.5 ± 7.8	89.0 ± 38.2	0.366
FVC%	104.00 ± 9.90	75.84 ± 64.39	0.601

**Table 2:** Comparison of postoperative results of the ICNB and c-SAPB groups. In bold values < 0.05.

	ICNB (#20)	C-SAPB (#20)	*p*
Block time (min)	2.53 ± 0.88	3.85 ± 2.52	0.091
VAS during chest tube removal	3.00 ± 1.15	1.95 ± 1.27	**0.022**
VAS 2 h after chest tube removal	0.81 ± 0.75	0.37 ± 0.68	0.080
Morphine request upon awakening (VAS > 4)	2 (10.0%)	5 (25.0%)	0.212
Morphine amount (mg)	0.77 ± 2.00	0.79 ± 1.78	0.960
Other drugs upon awakening	2 (10.0%)	1 (5.0%)	0.548
Paracetamol consumption I p.o.d. (mg)	1550.00 ± 760.88	2050.00 ± 759.16	**0.028**
Paracetamol consumption II p.o.d. (mg)	1650.00 ± 1190.97	1350.00 ± 1348.48	0.622
On-demand ketorolac consumption I p.o.d. (mg)	29.50 ± 23.05	30.00 ± 21.21	0.943
On-demand ketorolac consumption II p.o.d. (mg)	12.00 ± 11.85	10.25 ± 12.51	0.652
On-demand tramadol consumption I p.o.d. (mg)	25.00 ± 55.00	15.00 ± 32.85	0.490
On-demand tramadol consumption II p.o.d. (mg)	20.00 ± 59.69	2.51 ± 11.18	0.280
Chest tube length (days)	3.15 ± 1.09	2.90 ± 0.91	0.436
Chronic postsurgical pain	0	0	/
Complications block-related:			
Chest wall hematoma	0	0	/
Catheter dislodgement	/	0	/
Catheter discomfort	/	0	/
Nausea	0	0	/
Paresthesia	0	0	/
Other complications no block-related:			
Postoperative lung atelectasis	1 (5.0%)	0	0.311
Atrial fibrillation	0	0	/

**Table 3:** Comparison of postoperative results of the ICNB and c-ESPB groups. In bold values < 0.05.

	ICNB (#20)	C-ESPB (#20)	*p*
Block time (min)	2.42 ± 0.83	3.75 ± 1.23	0.130
VAS during chest tube removal	3.06 ± 1.21	2.35 ± 1.34	0.072
VAS 2 h after chest tube removal	0.78 ± 0.81	0.90 ± 0.97	0.677
Morphine request upon awakening (VAS > 4)	3 (15.0%)	5 (25.0%)	0.429
Morphine amount (mg)	0.30 ± 0.73	1.05 ± 1.93	0.113
Other drugs upon awakening	1 (5.0%)	0	0.311
Paracetamol consumption I p.o.d. (mg)	1800.00 ± 786.40	1750.00 ± 760.69	1.00
Paracetamol consumption II p.o.d. (mg)	2000.00 ± 1209.61	750.00 ± 1251.32	**0.005**
On-demand ketorolac consumption I p.o.d. (mg)	30.50 ± 19.86	32.25 ± 23.47	0.801
On-demand ketorolac consumption II p.o.d. (mg)	16.50 ± 13.38	8.25 ± 14.17	0.066
On-demand tramadol consumption I p.o.d. (mg)	30.00 ± 57.12	4.05 ± 15.36	**0.005**
On-demand tramadol consumption II p.o.d. (mg)	20.00 ± 69.58	0	**0.012**
Chest tube length (days)	3.40 ± 1.42	2.70 ± 0.86	0.339
Chronic postsurgical pain	0	0	/
Complications block-related:			
Chest wall hematoma	0	0	/
Catheter dislodgement	/	0	/
Catheter discomfort	/	0	/
Nausea	0	0	/
Paresthesia	0	0	/
Other complications no block-related:			
Postoperative lung atelectasis	0	0	/
Atrial fibrillation	0	0	/

**Table 4:** Comparison of postoperative results of the c-SAPB and c-ESPB groups. In bold values < 0.05.

	c-SAPB (#20)	C-ESPB (#20)	*p*
Block time (min)	3.81 ± 2.30	3.96 ± 1.65	0.789
VAS during chest tube removal	2.15 ± 1.23	2.26 ± 0.93	0.748
VAS 2 h after chest tube removal	0.55 ± 0.76	0.74 ± 0.81	0.461
Morphine request upon awakening (VAS > 4)	6 (30.0%)	5 (25.0%)	0.873
Morphine amount (mg)	1.00 ± 2.03	1.00 ± 1.71	1.00
Other drugs upon awakening	1 (5.0%)	1 (5.0%)	0.942
Paracetamol consumption I p.o.d. (mg)	1857.14 ± 792.83	1546.23 ± 904.83	0.225
Paracetamol consumption II p.o.d. (mg)	925.38 ± 1238.59	526.32 ± 1123.41	0.273
On-demand ketorolac consumption I p.o.d. (mg)	30.71 ± 19.25	26.05 ± 21.71	0.476
On-demand ketorolac consumption II p.o.d. (mg)	16.50 ± 13.38	6.32 ± 11.53	0.601
On-demand tramadol consumption I p.o.d. (mg)	11.67 ± 25.66	5.26 ± 22.93	0.413
On-demand tramadol consumption II p.o.d. (mg)	2.38 ± 10.91	0	**0.012**
Chest tube length (days)	2.95 ± 0.86	3.84 ± 3.72	0.293
Chronic postsurgical pain	0	0	/
Complications block-related:			
Chest wall hematoma	0	0	/
Catheter dislodgement	/	0	/
Catheter discomfort	/	0	/
Nausea	0	0	/
Paresthesia	0	0	/
Other complications no block-related:			
Postoperative lung atelectasis	0	0	/
Atrial fibrillation	0	0	/

## Data Availability

The data presented in this study are available upon request from the corresponding author.
